# Four Learner Categories in Global Health Experiences: A Framework for Successful Resident Engagement

**DOI:** 10.5334/aogh.3562

**Published:** 2022-08-08

**Authors:** Daniel A. Guiles, Edwin Nuwagira, Geren S. Stone

**Affiliations:** 1 Departments of Medicine and Pediatrics, Indiana University School of Medicine, Indianapolis, IN, US; 2 Department of Child Health and Pediatrics, Moi University School of Medicine, Eldoret, Kenya; 3 Department of Medicine, Mbarara University of Science and Technology, Mbarara, Uganda; 4 Department of Medicine, Massachusetts General Hospital, Boston, MA, US; 5 Department of Medicine, Harvard Medical School, Boston, MA, US

**Keywords:** Global health, medical education, global health experience, cultural humility

## Abstract

An increasing number of residency programs in the United States now offer global health experiences for trainees, yet many participating residents lack the behaviors and skills needed to engage effectively with local partners and colleagues. In the experience of the authors, trainees working in global settings fall into 1 of 4 learner categories determined by their degree of cultural humility and their willingness to engage with their hosts. This viewpoint proses the concept of “re-orientation,” or ongoing structured mentorship, as a way to provide key opportunities for residents to mature in these two important areas during their global experiences. We propose that residencies should incorporate “re-orientation” as a component of their global health rotations in order to provide their trainees with the skills and behaviors to engage successfully with their local colleagues and partners.

## Introduction

Interest in global health among medical trainees in the United States (U.S.) has increased exponentially since the turn of the 21^st^ century [1,2,3]. In 2019 alone, nearly one quarter of graduating medical students reported participation in a global health elective during their medical school years, and many have developed this interest further during their subsequent residency training [4]. With the increasing demand for global health training, numerous U.S. residency programs have formalized global health experiences into their curricula as electives, tracks, pathways, or even as dedicated residency programs [5,6]. These experiences possess the potential to benefit both U.S. and global partners, yet these experiences possess inherent potential for harm as well. They involve individuals from diverse cultural backgrounds, socioeconomic levels, and worldviews working closely together, which can lead to misunderstandings and conflict [1,6,7]. Residents who engage in humble and sensitive ways with their hosts can accomplish a lot of good for longitudinal partnerships, while those who do not can cause harm and limit the prospects for future collaboration [6,8].

In recent years, global health agencies and academic groups, such as the Consortium of Universities for Global Health (CUGH) and the Association of Schools and Programs in Public Heath (ASPPH), have developed standardized competencies to guide successful training in global health [9]. These competency-based models have been further discussed in a recent review article by Schleiff et al., which, among other items, recommends that programs seek to focus on “soft skills” such as leadership, communication, and cultural competency when training future global health professionals [9]. Many U.S. residency programs have sought to accomplish this through a variety of educational activities, including pre-departure orientations, global health learning modules or curricula, and post-rotation debriefing sessions (summarized in Table 1) [1,2,3,8]. It is the experience of the authors that these activities, while important and helpful, are not enough to guarantee humble and sensitive engagement by the resident with their local colleagues and patients, particularly in these “soft skill” areas. One key determining factor is the individual trainee’s underlying set of assumptions about themselves and their hosts, which often do not become apparent until residents are on the ground participating in their global health experience. Trainees are often unaware of their assumptions and do not possess the skills and behaviors needed to identify them, and to thus engage effectively with their local colleagues. This means that predeparture training and orientation are often not sufficient in identifying and addressing the ongoing needs of trainees. While post-rotation debrief sessions may serve to identify some areas of struggle, they have the disadvantage of occurring after the rotation is complete, meaning that opportunities for addressing challenging behaviors or assumptions in the moment are missed.

**Table 1:** Global Health Rotation Training Summary.


PRE-ROTATION TRAINING	GLOBAL HEALTH ROTATION	POST-ROTATION DEBRIEFING

Global health curricula	Post-arrival orientation	Written Reflections

Pre-departure orientation to site	Post-arrival cultural sensitivity training	Face-to-face meetings with U.S. based supervisors

Cultural sensitivity training	Post-arrival safety and security briefing	Scholarly output (presentations, posters, etc.)

Ethics Training/simulations		Formal feedback mechanism (survey, evaluations) used to evaluate the program

Pre-departure safety and security briefing		


**Figure 1:**
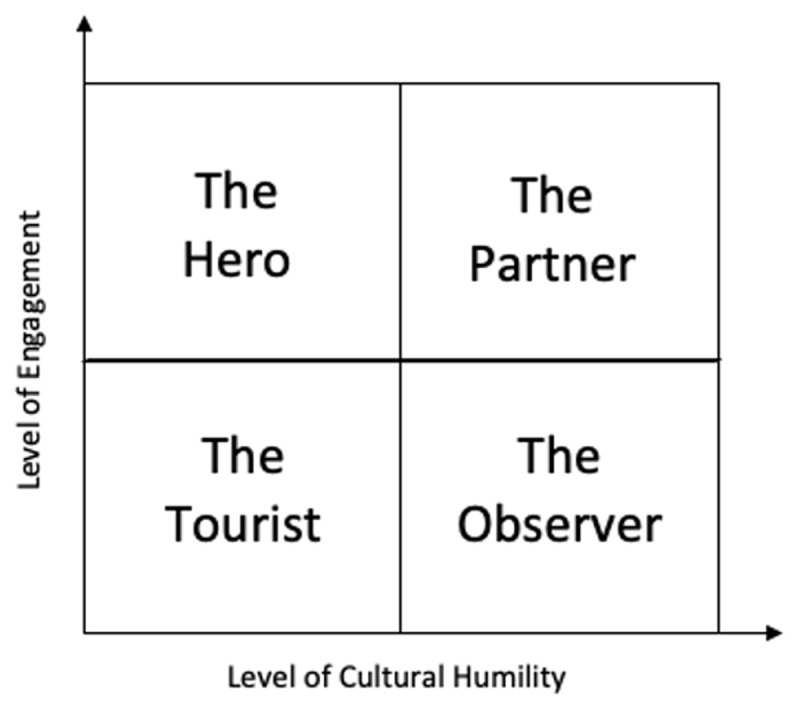
Four learner categories in global health experiences.

In the experience of the authors, most cross-cultural misunderstandings in global health rotations result from deficiencies in two main areas: 1) the resident’s willingness to engage with their local colleagues and health system, and 2) the degree of cultural humility exhibited by the resident as they seek to engage with the local health system. “Cultural humility” has been defined as a lifelong commitment to self-evaluation and critique with the goal of recognizing and breaking through beliefs, assumptions and stereotypes that can get in the way of being appropriate or sensitive in another’s culture [10]. Individual learners engaging in cross-cultural experiences are generally at different stages of development in these two areas and, in our experience, tend to fall into 1 of 4 specific learner categories (summarized in Figure 1): The Tourist, The Hero, The Observer, and The Partner (which is the ideal learner category). Each of the first three categories has its own struggles and blind spots that lead to specific patterns of misunderstanding and conflict between learners and their local partners. By recognizing where a particular learner falls within this framework, supervisors can anticipate areas of conflict, pre-emptively address them, and mentor the trainee toward more effective engagement patterns. It is the view of the authors that this is best achieved through ongoing structured mentorship activities occurring at regular intervals throughout the global health experience.

## The Four Learner Types

The following is a brief description of each of the four learner types:

**The Tourist:** As demonstrated in Figure 1, trainees in this category score low in both the cultural humility and engagement domains. They generally demonstrate great interest in global health experiences, yet their enthusiasm directed more toward the opportunities that working in new settings can provide for tourism, or for developing their own personal brand. Learners in this category are readily identifiable by their superficial interactions with their local colleagues and by their limited level of engagement with the local health and educational systems. They lack active participation in patient care and hesitate to work with their local counterparts in academic or other non-clinical activities. When asked about their rotation they are likely to report sensational stories related to the challenges encountered with the local health system or to limit their discussion to sightseeing activities they have participated in. They are far less likely to mention lessons learned from their local team members or how they have grown as physicians and as individuals during their rotation. In summary, they are primarily focused on their own experiences (the more exotic the better) and on the stories (medical or otherwise) that can be shared or used to impress colleagues, friends and/or family.

**The Hero:** Learners in this category score high in engagement, but low in cultural humility. They approach their global health elective with a strong desire to participate yet fail to do ways that acknowledge their own limited assumptions and blind spots. Many have limited experience with navigating healthcare delivery in new settings, and struggle to adapt to clinical situations that are handled differently from how they would be at their home institutions. Heroes generally possess an inflated view of their own level of medical knowledge when compared to that of their local counterparts, and struggle to understand the reasons for the differences they are experiencing. Meaningful relationships can be incredibly challenging for this learner type, and much damage can occur if their behaviors go unaddressed. These can range widely, from the learner making condescending and rude remarks, to performing heroic but potentially harmful activities for very sick and complicated patients. These learners fail to perceive the potential harm of these actions to their local colleagues, patients, and their families. Out of all the learner types, this one tends to be the most damaging.

**The Observer:** Observers score high in humility, but low in engagement, which results in an overly apprehensive posture. Many global health experiences involve new health systems and new diseases, making it challenging for learners in this category to know exactly how they should engage with their host health system, particularly if their role is not well defined. Expectations for trainees at various levels of training can also be quite different in their new setting, and it can take some time to figure out what roles a resident should perform within a particular healthcare team. Observers respond to this by being hesitant and withdrawn, which greatly affects their daily work alongside their local colleagues, and results in an inability to contribute to patient care decisions. This failure to actively participate can be extremely off-putting to local colleagues and supervisors who may view the trainee as disinterested or uncaring. This lack of willingness to engage does not allow for meaningful interactions between residents and their local colleagues and severely limits the value of the rotation for both parties.

**The Partner:** The Partner represents the ideal learner category to which all others should aspire. Partners score highly in both cultural humility and engagement and view their global health experience as a unique opportunity to learn from a healthcare setting that is different from their own. They continually seek to identify their limitations and to challenge their assumptions about themselves and others. Partners not only have a strong desire to learn from their hosts, however. They also recognize that they bring unique knowledge, skills, perspective, and experience to the table, and that these can be of value to their local counterparts. They possess a willingness to work side by side with colleagues, and an attitude that encourages trust, friendship, and respect.

## “Re-Orientation” in Resident Global Health Experiences

Ideally, all residents who participate in global health rotations would fall into the “Partner” category; however, in practice this is often not the case. It can be challenging for supervising faculty and program leadership to accurately predict which category will correspond to a particular resident until they are on the ground participating in the global elective. In order to provide adequate and timely mentorship for trainees and to set them up for success, we propose incorporating structured mentorship activities into the global health elective, which we have termed “re-orientation”. We define “re-orientation” as structured mentorship sessions, occurring at regular intervals throughout the global health experience, that serve to provide trainees with opportunities to discuss, identify, and address their harmful behaviors and assumptions. The goal is to identify those residents in the first three learner categories (Tourists, Heroes, and Observers) and to nudge them toward the “partner” learner category. This is accomplished by reinforcing effective engagement strategies and by encouraging a posture of cultural humility.

“Re-orientation” can take on a variety of formats but should always involve regular, scheduled time set aside for guided self-reflection and feedback. It needs to be a safe space where residents are allowed to ask difficult questions and blow off steam, but ultimately should help residents move beyond their struggles to how they will seek to address them moving forward. Faculty facilitators, either from home or host institutions, should seek to gently correct misconceptions and encourage residents to identify any biases or stereotypes that may be limiting their ability to participate fully with their local counterparts. These sessions should ideally take place in person, either individually or in smaller groups. However, programs that do not possess on site faculty could conceivably accomplish this through scheduled calls and videoconferencing or, if these are impractical due to scheduling and time differences, by incorporating written reflections that allow for timely written feedback from supervisors. They should occur throughout the rotation, in order to encourage the trainee to make changes in the moment, and before the end of the experience. While open ended questions are helpful to encourage self-reflection and group discussion, the sessions should always conclude with concrete steps for how the learner will apply lessons learned for the remainder of their rotation (see Table 2 for an example of the types of discussion questions that can be used). “Re-orientation” will look different depending on an individual’s learner type (whether tourists, heroes, observers, or partners), but it will always involve encouraging each trainee to mature in areas of effective engagement and cultural humility. Ultimately, it should provide opportunities for residents to process their experiences, reflect on their behaviors (and those of their patients and local colleagues) and identify successes and failures. By the end of each session, trainees should be able to better understand the underlying reasons for misunderstandings or situations of conflict and should have identified concrete ways to move closer toward the Partner learner category.

**Table 2:** Re-orientation Example Format.


RE-ORIENTATION QUESTION	DESCRIPTION

“Tell me how your week has gone so far.”	Encourage trainee(s) to bring up key experiences to help guide the discussion.

“What clinical activities have you been involved with? What academic activities? How have those gone?”	Assess their level of engagement in clinical and educational activities.

“What successes or positive experiences have you had this week?”	Assess how they are defining “success” as they engage with the experience.

“What challenges have you faced this week?”	Assess how they are handling these challenges. Are they doing so in ways that demonstrate humility and a willingness to learn?

“How are things with your team? What is your role?”	Assess level of engagement with their local colleagues and level of cultural humility.

“Any questions or concerns about patient care?”	Assess if they are challenging their own biases and assumptions and seeking to understand before passing judgment.

“Any questions or concerns about your team?”	Assess if they are challenging their own biases and assumptions and seeking to understand before passing judgment.

“What feedback did you receive this week from your team or local supervisor?”	Encourage trainee to actively seek and humbly reflect on feedback from colleagues and local supervisors.

“Any questions or concerns how the rotation is going?”	Cover any other relevant concerns or issues.

“What lessons have you learned that you will apply in the coming days?”	Encourage trainee to come up with practical ways to engage more effectively and/or with cultural humility.


## Conclusion

Global health experiences are invaluable experiences for U.S. medical residents, yet they are often beset by challenges that require large amounts of nuance and patience. Learners participating in these rotations take with them assumptions and behaviors that are often limited and incorrect, which can make it challenging for them to engage effectively with their hosts. In our experience, trainees tend to fall into one of four categories (Tourists, Heroes, Observers, and Partners) depending on their level of cultural humility and their ability to engage effectively with their local counterparts. We suggest that residency programs can better equip their trainees for effective global health experiences by recognizing these learner categories, and by providing ongoing structured mentorship opportunities for them to reflect on and address their biases and misconceptions.
